# Rats distinguish between absence of events and lack of evidence in contingency learning

**DOI:** 10.1007/s10071-012-0524-8

**Published:** 2012-06-29

**Authors:** Michael R. Waldmann, Martina Schmid, Jared Wong, Aaron P. Blaisdell

**Affiliations:** 1 Department of Psychology, University of Göttingen, Gosslerstr. 14, 37073 Göttingen, Germany; 2 Department of Psychology, University of California, Los Angeles, CA USA

**Keywords:** Event representation, Contingency learning, Extinction, Renewal

## Abstract

The goal of three experiments was to study whether rats are aware of the difference between absence of events and lack of evidence. We used a Pavlovian extinction paradigm in which lights consistently signaling sucrose were suddenly paired with the absence of sucrose. The crucial manipulation involved the absent outcomes in the extinction phase. Whereas in the Cover conditions, access to the drinking receptacle was blocked by a metal plate, in the No Cover conditions, the drinking receptacle was accessible. The Test phase showed that in the Cover conditions, the measured expectancies of sucrose were clearly at a higher level than in the No Cover conditions. We compare two competing theories potentially explaining the findings. A cognitive theory interprets the observed effect as evidence that the rats were able to understand that the cover blocked informational access to the outcome information, and therefore the changed learning input did not necessarily signify a change of the underlying contingency in the world. An alternative associationist account, renewal theory, might instead explain the relative sparing of extinction in the Cover condition as a consequence of context change. We discuss the merits of both theories as accounts of our data and conclude that the cognitive explanation is in this case preferred.

## Introduction

The coordination between subjective experiences and mental representations of the world is a daunting task that all intelligent organisms face. Experiences only provide incomplete information about the current state of the world and are notoriously ambiguous. One example comes from object permanence research, in which objects are first shown to children and then are moved behind an occluder (Piaget 1937/1954). In this situation, children have a choice between assuming that the object has suddenly ceased to exist or that it has just gone out of sight for the time being.

A second example, which has received scant attention and is the focus of the current experiments, involves contingency learning. In contingency learning, we do not track individual objects (such as in object permanence studies) but pick up statistical regularities between types of events. For example, one of Pavlov’s dogs (Pavlov 1927) may have learned that tone signals are regularly followed by food presentations. Now imagine that the contingency suddenly seems to have changed, with continued tone signals but no access to food (what Pavlov termed *extinction*). This creates an ambiguity the dog must resolve. Either the world has changed so that the new contingency reflects a change of the underlying causal structure, or the world has not changed but the dog somehow missed the food. If the dog assumes that the world typically contains stable causal relationships, then the change of the experienced contingency needs to be attributed by the dog to the information that reached its senses rather than to a change of contingencies in the world. Assuming causal stability, the dog might conclude that it has simply not seen the food because it was hidden or occluded from sight.

In the present studies, we investigated the capacity to resolve ambiguities in contingency learning in rats, a species whose learning processes are, in the literature, predominantly modeled by associative theories. Traditional behaviorist theories of associative learning were ill-equipped to resolve the described ambiguities in the informational input. Events were typically coded as present or absent with associations directly reflecting experienced contingencies (e.g., Rescorla and Wagner 1972). Distinguishing between experienced contingencies and contingencies in the world is beyond the representational assumptions of traditional stimulus–response (S–R) theories and was largely ignored in Pavlov’s (1927) seminal work on stimulus–stimulus (S–S) learning.

In the past decades, the focus in associative theorizing has changed from a behaviorist S-R perspective to a more cognitive focus on mental representations of learning events that take part in learning. Currently, classical conditioning is in most cases modeled as involving associations between mental representations of the world (S–S learning; see Delamater and Oakeshott 2007; Holland 1990, for an overview). According to this view, cues (e.g., tones) may elicit images of predicted outcomes (e.g., food), which in turn may elicit visible responses. Moreover, cues may also activate images of each other (Larkin et al. 1998) or may acquire perceptual processing of outcomes in their absence (Holland 1990).

Allowing images to be part of learning processes dramatically increases the representational power in learning but creates the problem of reality monitoring (see also Holland 1990). How does a learning system operating on images decide whether the relations between the images reflect reality, or are just illusory correlations? If in extinction learning, for example, the cues are followed by images of the outcome, then how is it possible that an organism learns that the underlying contingency has changed?

To avoid insensitivity to the changes in the world, the learning system needs to be able to compare the elicited images to the real events, and distinguish between events and images. Holland (1990) reported evidence showing that rats indeed distinguish between images and events. This distinction is fairly easy when the elicited images can be directly compared to experiences of present events (e.g., food). When no sensory information about the presence of the event is available, however, the organism needs to decide whether the event is actually absent or whether the current information about the event is inconclusive because perceptual access to the event is blocked.

### Experimental task and conditions

In the following experiments, we investigated whether rats are sensitive to the difference between absence of events and lack of evidence. We approached this question using a conventional Pavlovian extinction paradigm. In the two crucial experimental conditions, in Phase 1, the Acquisition phase, rats learned over several days about a perfect contingency between a cue, a light signal, and sucrose in the receptacle of a Skinner box. In Phase 2, the contingency was changed to zero (i.e., extinction) for the two groups of rats. Sucrose was always absent regardless of the presence or absence of the light cue. In Phase 3, the Test phase, the cues were presented without sucrose again, and we measured nose pokes for sucrose in the receptacle.

**Fig. 1:**
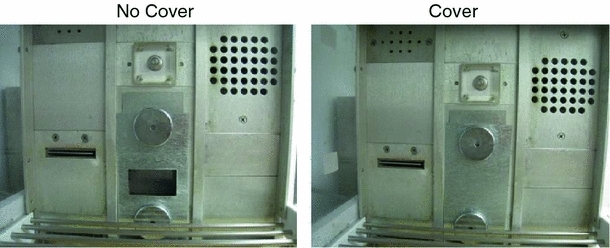
Pictures of the apparatus configurations in the No Cover (*left panel*) and Cover (*right panel*) conditions. In the Cover condition, a metal plate blocked access to the drinking receptacle

The crucial manipulation involved Phase 2 that contrasted two conditions. In Experiments 1 and 2, in all conditions, a metal plate was attached on the receptacle throughout the experiment (see Fig. 1). In the No Cover condition, a small opening provided access to the receptacle (Fig. 1, left panel). Thus, in this condition, the rats could directly experience that sucrose was absent during the extinction phase. Additional control conditions were also included in the experiments.

### Two competing theoretical accounts

In recent years, theories that grant more sophisticated cognitive competencies to rats than traditional associationist models have become increasingly popular (see Blaisdell and Waldmann 2012). A cognitive account would predict for our tasks that in the No Cover condition, rats should learn in the Extinction phase that the contingency has changed, which should manifest itself in a decrease of search time in the Test phase. By contrast, in the Cover condition, a metal plate blocked access to the receptacle during extinction, thereby preventing the rats from checking whether sucrose was present or absent behind the plate (Fig. 1, right panel). As in the No Cover condition, the presence of the light cue was followed by the absence of the experience of the sucrose outcome. If the rats did not distinguish between absence (No Cover) and lack of evidence (Cover), rats in both conditions should show low expectancies (i.e., extinction). On the other hand, if rats understand that the metal plate blocks access to information about the sucrose, they should understand that in Phase 2, the contingency between the light and sucrose might still be intact despite the fact that the metal plates make it impossible to monitor this contingency. Of course, the contingency information is ambiguous; it could also be the case that the contingency has changed behind the cover. If the rats assume that causal relations in the world are typically stable, the conclusion that the contingency is intact seems more plausible, but should be tempered with a certain degree of uncertainty.

However, there is a popular competing associationist account that makes similar behavioral predictions but does not require the attribution of sophisticated cognitive representations. According to *renewal* theory, the differences between the Cover and No Cover conditions may be due to the fact that in the Cover condition, a context change has taken place (Bouton and Bolles 1979). In a typical renewal paradigm, a conditioned response is established in Context A, whereas extinction takes place in Context B. In this paradigm, it is typically found that rats and other organisms, when tested in Context A again, show an immediate return of the conditioned response, the renewal effect. Apparently, extinction is bound to its specific context and does not generalize to the original learning context. One popular explanation of this pattern is that context plays the role of an occasion setter for the learning and extinction episodes (see Bouton 2004; Bouton and Bolles 1979; Delamater 2004). Thus, on the view that the introduction of the intact metallic shield in the Cover condition constitutes a salient context change, it can be argued that extinction should be diminished in the Test phase because extinction training occurred in a context that was different from both the learning and the test conditions.

Initial research on the renewal effect made context change as salient as possible (e.g., different rooms), which also makes it plausible on a cognitive account that learners may be aware of the specificity of the learning and extinction contexts. However, more recently, it has been shown that subtle differences in context show renewal effects. Thomas et al. (2003), for example, found that small changes of odor, location within a room, or unintended differences between Skinner boxes suffice to create renewal effects. Bouton (2004) reports various studies that show that renewal effects can also be obtained when organisms had ingested different drugs during different phases of the learning sessions. Thus, on the basis of these findings, the presence or absence of a metallic cover could very well constitute a relevant context change according to renewal theory.

How can the two theoretical paradigms be tested against each other? One problem of testing our cognitive account against renewal theory is that renewal theory is more general, so that any behavioral predictions by a cognitive account can be mimicked by renewal theory. Given that any manipulation of cognitive representations across different phases of the extinction paradigm requires us to change aspects of the stimulus characteristics of the task, it will always be possible for a renewal theorist to postulate post hoc that the chosen changes in the stimuli and the task constitute a relevant context change. And if the data do not show a renewal effect, it can be argued post hoc that the context change was not salient enough to affect learning. Thus, from a post hoc perspective, it is impossible to prove that renewal theory is inadequate. Although this flexibility of renewal theory may look like a strength, it is also a weakness. Whereas our cognitive account makes clear *predictions* about relevant and irrelevant context changes, it is less clear how renewal theory can predict which one of otherwise similar context changes will exhibit a renewal effect.

We will capitalize on this weakness by testing our theory in a paradigm in Experiment 3, in which we implemented superficially similar context changes in both the Cover and the No Cover conditions. In both conditions, the metallic plate was only present in the Extinction phase, but not shown in the other three phases (i.e., magazine training, Acquisition training, and Test phase). Thus, in both experimental conditions, similar context changes were implemented. Moreover, in both conditions, we used the same intact metallic plate. The crucial manipulation was that in the No Cover condition, the metallic shield was slightly moved a couple of inches away from its usual position on the receptacle, so that it did not obstruct the opening anymore. According to our theory, this should make a huge difference, but on an account that is sensitive only to context change, it is unclear how it can be justified that in Experiment 3 the context changes in the Cover condition should be relevant, whereas the context change in the No Cover condition should not. Without implicitly using a cognitive interpretation of the task, it is hard to see how a theory devoid of the representational resources of cognitive theories can justify such a prediction (see also “General discussion”).

## Experiment 1

The main goal of this experiment was to provide initial empirical evidence for our prediction that extinction should be sensitive to the presence or absence of a cover hiding the drinking receptacle. In the experiment, rats first learned a contingency between a light signal and sucrose and then underwent an extinction phase, in which sucrose was generally absent. The crucial manipulation was whether a full cover (Cover condition) was placed over the receptacle, or a cover that left a small opening (No Cover). We predicted that the extinction phase will have less influence on the search behavior in the Test phase for rats in the Cover than in the No Cover condition, although, due to the ambiguity of whether sucrose is present or absent behind the cover, there may still be a small tendency toward some extinction in the Cover group.

### Subjects

Sixteen experimentally naïve female Long-Evans rats (*Rattus norvegicus*) acquired from Harlan (Indianapolis, IN) served as subjects. Subjects were pair-housed in transparent plastic tubs with a wood shaving substrate in a vivarium maintained on a 12-h light/dark cycle. Experiments were conducted during the dark portion of the cycle. A progressive food restriction schedule was imposed prior to the beginning of the experiment, which allowed us to maintain rats at 85 % of their initial free-feeding weights. All animals were handled for 45 s during the 3 days prior to the initiation of the study. Subjects were randomly assigned to one of two groups (*n*s = 8): Cover and No Cover.

### Apparatus

The experiment was conducted in a small room containing eight Skinner boxes. Each Skinner box measured 30 × 25 × 20 cm (*L* × *W* × *H*) and was housed in a separate sound- and light-attenuating environmental isolation chest (ENV-008, Med Associates, Georgia, VT). The front and back walls and ceiling of the chamber were constructed of clear Plexiglas, the side walls were made of aluminum, and the floors were constructed of stainless steel rods measuring 0.5 cm in diameter, spaced 1.5 cm center-to-center. The enclosure was dimly illuminated by a 28-V bulb (ENV-215 M, Med Associates) house light located 2 cm from the top of the left-side chamber wall house light.

Each chamber was equipped with a water-dipper (ENV-202 M, Med Associates) that could be lowered into a trough of sucrose solution (20 % by volume) and raised. When in the raised position, a small well (0.05 cc) at the end of the dipper arm that contained sucrose solution protruded up into the drinking receptacle. Delivery of the sucrose solution served as the appetitive outcome (called an Unconditioned Stimulus or US in conventional Pavlovian terminology). The drinking receptacle could be covered with one of two types of stainless steel metal plates (each 12.5 cm high × 6.5 cm wide) that were held in place by two 3.5-cm-diameter circular magnets (each 15 lbs. force), one placed above and one placed below the receptacle opening (see Fig. 1). One type of metal plate had a 2.5 cm high × 4.4 cm wide opening (the bottom edge of the opening was flush with the bottom edge of the drinking receptacle). This opening allowed access to the drinking receptacle (Fig. 1, left panel). The other type of metal plate was completely solid, so when in place it prevented physical or visual access to the receptacle (Fig. 1, right panel). A diffuse light (ENV-227 M, Med Associates) was located 2 cm from the top of the right-side chamber wall, directly above the opening of the drinking receptacle, and when flashed at a rate of 2 (on–off) cycles per second served as the conditional stimulus (CS) (i.e., the signal or cue). Ventilation fans in each enclosure and a white noise generator on a shelf outside of the enclosures provided a constant 62-dB(A) background noise.

The chamber in which training, extinction, and testing took place stayed the same for each rat. Although not all 8 subjects of each condition were run at the same time, each rat was run at the same time of the day. Moreover, the time of day was controlled for by way of counterbalancing—we ran 2 squads of 8 rats, and each squad consisted of subjects from all groups.

We measured expectation of sucrose by monitoring feeder activity in the receptacle where sucrose was delivered during training. Whenever the rat placed its nose into the receptacle (a nose poke), it disrupted an infrared photobeam projected across the entrance to the hopper. Nose pokes were defined as the amount of time (every 100 ms) that rats broke the infrared beam in the drinking receptacle with their noses.

### Procedure

#### Magazine training

Day 1. During this phase, the drinking receptacle in each chamber was covered with a metal plate that had an opening to allow access. Rats could explore the box and drink from the dipper containing sucrose, which delivered sucrose solution every 20 ± 15 s (actual ITI values = 5, 10, 15, 20, 25, 30, and 35 s, selected randomly). The familiarization phase lasted 60 min; sucrose was delivered independently of the rats’ behavior. The number of nose pokes was used as an indicator of learning.

#### Phase 1: acquisition

**Fig. 2:**
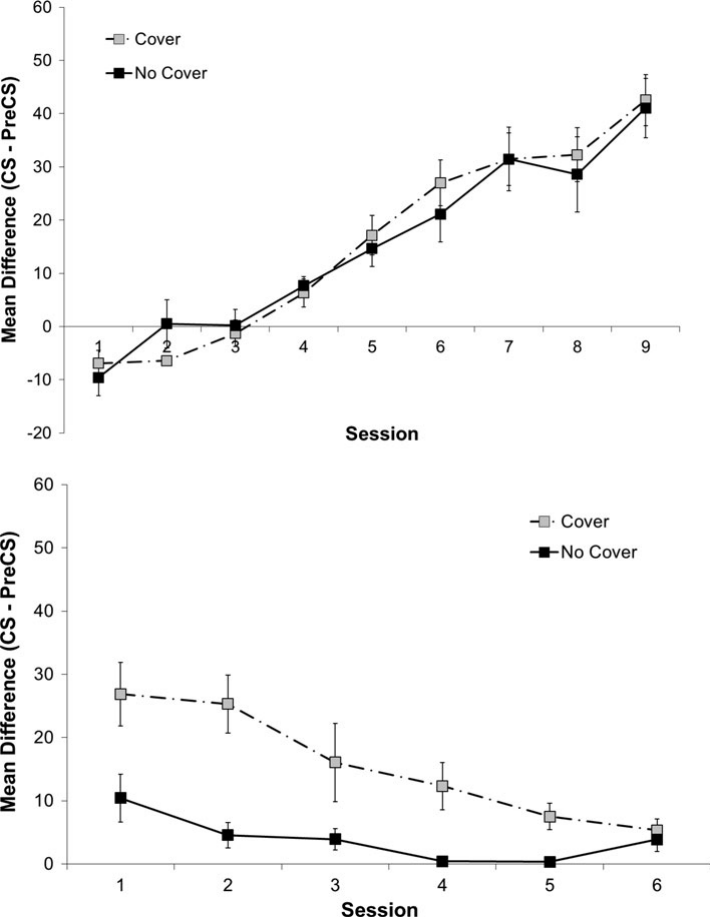
(*Top panel*) Mean difference (CS rate per 10 s—pre-CS rate per 10 s) scores (discrimination index) as a function of Phase 1 Acquisition session for the Cover and No Cover conditions in Experiment 1. *Error bars* represent standard errors. (*Bottom panel*) Mean difference (CS—pre-CS) scores (discrimination index) as a function of Phase 3 Test session for the Cover and No Cover conditions in Experiment 1. *Error bars* represent standard errors

Days 2–10. During this phase, the drinking receptacle in each chamber was covered with a metal plate that had an opening to allow access to the receptacle (Fig. 1, left panel). All rats received 2 light-sucrose pairings per 60-min session on the first 2 days and then 4 pairings on the remaining days. The difference was due to a software error on the first 2 days. On each trial, the light was presented for 10 s followed immediately after it terminated with a 10-s presentation of sucrose. Trials were delivered with a mean interval of 15 ± 3 min (actual ITI values were 8 and 34 min on the two trial days, and 12, 18, 18, and 12 min on the days with four trials). We recorded all nose pokes (i.e., breaks in the photobeam) emitted during the light (i.e., 10 s) and during a 30-s period prior to the onset of the light. The 30-s pre-CS/10-s CS measure is a convention commonly adopted by our and other laboratories. To assess learning, we calculated discrimination indices to measure acquisition of excitatory responding for sucrose during the light (i.e., an increase above baseline in time spent nose poking). Discrimination indices were calculated as difference scores between pre-light and light nose-poke responding. Since we measured pre-light responding for 30 s, we divided this value by 3 to obtain an averaged value that corresponds to the 10 s of CS responding. Thus, if we report an “increase above baseline” value of 25, for example, it will mean that on average subjects made 25 nose pokes per 10-s CS period above and beyond the rate that they did during baseline (pre-CS). One rat from the No Cover group showed lower rates of nose poking by Day 9 of Phase 1 relative to the other subjects (i.e., discrimination ratio below 0.8) and therefore received an additional Acquisition session on Day 10 (extra session not shown in Fig. 2).

#### Phase 2: extinction

Days 11–13. Prior to Phase 2, for rats in Group Cover, the drinking receptacle was covered with a metal plate that did not have an opening, thereby preventing access to the receptacle (Fig. 1, right panel). Although the dipper was again lifted in this condition (no noticeable vibrations for the human ear), with the metal plates in place the rats could not investigate the drinking receptacle, and thus could not visually determine the status of the sucrose dipper. For rats in the No Cover group, the drinking receptacle in each chamber was covered with a metal plate that had an opening to allow access to the receptacle (Fig. 1, left panel). Again, the dipper was lifted but did not contain sucrose. Thus, rats in the Cover and No Cover groups received 12 presentations of the light alone in each 30-min session, with a mean intertrial interval of 2.5 min (i.e., 1, 2, 3, and 4 min, randomly picked). For subjects in the No Cover condition, we recorded all nose pokes emitted during a 30-s period prior to the onset of the light and during the presentation of the light. This allowed us to calculate a discrimination index for this group, which serves as a measure of excitatory responding for sucrose during the light. Similar measures could not be made for subjects in the condition with a cover.

#### Test phase

Days 14–19. During this phase, the drinking receptacle in each chamber was covered with a metal plate that had an opening to allow access to the receptacle (Fig. 1, left panel). In each 35-min test session, rats received 4 test trials with the light but no sucrose. These test presentations occurred at 8, 16, 24, and 32 min into the session. We recorded all nose pokes emitted during each 30-s interval prior to the onset of the light and during each presentation of the light. No sucrose was delivered during these sessions but, as in prior phases, the (empty) dipper was activated after each test presentation of the light.

### Results and discussion

Figure 2 (top level) shows the nine sessions (extra session for the slow learners not shown) on Days 2–10. The rats acquired behavioral control by the light-sucrose contingency equally well regardless of condition (see Fig. 2, top panel). Since sphericity cannot always be assumed in our data (Mauchly’s test of sphericity, *p* < 0.0001), we used the Greenhouse–Geisser correction to analyze the within-subjects tests for both the acquisition and test data of experiments, whenever necessary. An analysis of variance using the difference between pre-light and the light nose-poke responding as an indicator of learning (i.e., discrimination index) only showed an effect of day of learning session, *F*(3.43, 48.05) = 41.18, *p* < 0.001, MSE = 280.42, but there was no significant difference between conditions nor an interaction effect.

Figure 2 (bottom panel) shows the results of the Test phase for which expectancy of sucrose was measured subsequent to the extinction training (6 sessions on Days 14–19). An analysis of variance conducted on discrimination difference scores as a measure of expectancy showed decreasing values across test sessions, *F*(2.51, 35.14) = 9.89, *p* < 0.001, MSE = 114.62. The decrease reflects further extinction within the Test phase (which actually embodies further extinction treatment). Importantly, there was a clear difference between conditions, *F*(1, 14) = 13.64, *p* = 0.002, with a significant Condition × Session interaction, *F*(2.51, 35.14) = 3.19, *p* = 0.043. A post hoc test comparing the difference in rates of extinction (defined as slope) between the Cover and No Cover conditions revealed a significant effect, *t*(14) = −2.64, *p* = 0.02, with the Cover condition showing a steeper decline (*M* = −4.71, SEM = 1.11) than the No Cover condition (*M* = −1.40, SEM = 0.58). Further analyses with the Bonferroni adjustment for multiple comparisons of difference scores within the first and the last test sessions revealed a significant difference between the Cover (*M* = 26.86, SEM = 5.02) and the No Cover (*M* = 10.44, SEM = 3.76) conditions for the first test session, *t*(14) = 2.62, *p* = 0.02. No such difference was found for the final test session, demonstrating a convergence in scores (Cover condition: *M* = 5.34, SEM = 1.81; No Cover condition: *M* = 3.87, SEM = 1.87).

In sum, on our cognitive account, the results confirm our expectation that rats understood the difference between absence and lack of evidence. When a cover blocked access to the drinking receptacle during extinction training, the expectancy of sucrose during light signals remained fairly high in the Test phase as compared to the No Cover group that received conventional extinction treatment by having access to the drinking receptacle. Although, relative to the end of Acquisition training, some extinction was evident in all groups in the Test phase, the uncertainty created by the cover appeared to protect the rats in the Cover condition from complete extinction.

Alternatively, on an associationist account, the results may be interpreted as evidence for an effect of context change across the phases (i.e., renewal). It is important to note that we already tried to weaken the plausibility of this account by constantly presenting a metallic plate in all conditions, so that context change was minimal. However, since the cover needed to be modified in the Cover condition to completely cover the receptacle, it is possible that this modification gave rise to a renewal effect. In Experiment 3, we will re-visit the question of how the two competing accounts can be empirically distinguished.

## Experiment 2

In Experiment 2, we pursued two aims. First, we wanted to replicate the effect between the Cover and No Cover conditions with a larger sample and different treatment parameters. Second, we included a control condition that allowed us to assess the degree of extinction in the Cover and No Cover conditions relative to a baseline of no extinction. Thus, in the new Acquisition Control condition, we simply continued contingency treatment in Phase 2 rather than giving the rats’ extinction training.

### Subjects and procedure

There were three groups, with 11 female Long-Evans rats in the Cover, 11 in the No Cover, and 10 in the Acquisition Control conditions. The equipment and procedure were the same as for Experiment 1 except where noted below. All rats received 60 min of magazine training, in which they were exposed to sucrose solution from the drinking receptacle. The same ITIs as in Experiment 1 were used. As in the previous study, nose pokes were measured as indicators of expectancies. Two subjects that showed a low amount of licking were given additional magazine training sessions. Phase 1 Acquisition training was the same as in Experiment 1 except that all rats received only six consecutive days of Phase 1 treatment. In the previous experiment, we had already obtained performance near the ceiling after Session 6. Each subject received three light-sucrose pairings within each daily 30-min session. On average, the intertrial interval lasted 10 min (actual ITI values were 9, 10, and 11 min, randomly picked). In Phase 2, the three conditions differed again. Phase 2 lasted 4 days, with 12 trials in each 30-min session. Thus, we ran one more day of extinction than in Experiment 1 to equate levels of performance between the two experiments, which for unknown reasons took longer in Experiment 2. Phase 2 treatment for Groups Cover and No Cover were as described for Experiment 1. The group Acquisition Control received continued exposure to the light-sucrose contingency in Phase 2 with 12 light-sucrose pairings. The Test phase was similar to that of Experiment 1. Again the empty dippers inside the receptacles were accessible to all rats following each test trial. All rats received three trials per 30-min session with the same intertrial intervals as in Experiment 1. Testing continued for 6 days until performance in the three conditions merged (i.e., tested to extinction).

### Results and discussion

**Fig. 3:**
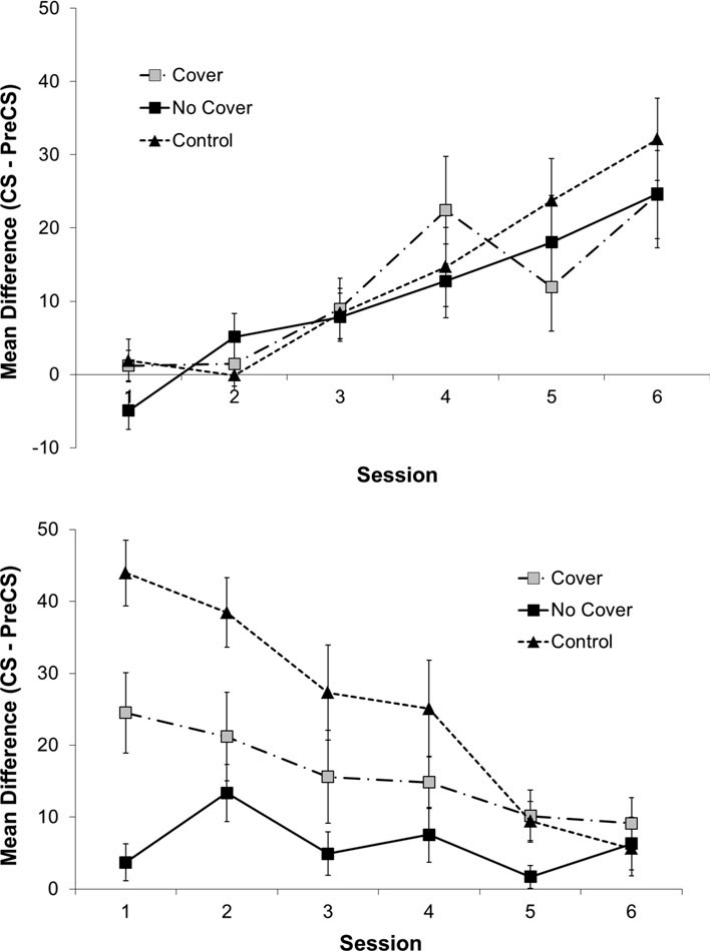
(*Top Panel*) Mean difference (CS—pre-CS) scores (discrimination index) as a function of Phase 1 Acquisition session for the Cover, No Cover, and Control conditions in Experiment 2. *Error bars* represent standard errors. (*Bottom panel*) Mean difference (CS—pre-CS) scores (discrimination index) as a function of Phase 3 Test session for the Cover, No Cover, and Acquisition Control conditions in Experiment 2. *Error bars* represent standard errors

Figure 3 (top panel) shows the results from the Acquisition phase as measured by discrimination indices (difference scores). The contingency between light signals and sucrose were uniformly acquired in all three conditions, *F*(3.65, 105.74) = 19.88, *p* < 0.0001, and MSE = 244.13. There was no significant difference between groups nor a significant interaction. We additionally analyzed learning performance of the Acquisition Control condition in the sessions that corresponded to extinction training in the Cover and No Cover conditions. Recall that the control animals received additional learning trials in this period. The analyses show that relative to the last session of the regular Acquisition training sessions, no signs of further learning can be seen in the behavior. Thus, by Session 6, the ceiling has been already reached with no additional increases in the four following sessions, *F*(4, 50) = 0.223, *p* = 0.925.

In the Test phase, we replicated the statistical difference between the experimental conditions from the last experiment (Fig. 3, bottom panel). An analysis of variance on the discrimination difference score as the dependent measure showed a decreasing trend across test sessions, *F*(3.73, 108.15) = 11.14, *p* < 0.0001, MSE = 225.72, which signifies further extinction within the Test phase. More importantly, there was a clear difference between the conditions, *F*(2, 29) = 10.81, *p* < 0.0001, which was moderated by a significant interaction, *F*(7.46, 108.15) = 3.43, *p* = 0.002. Planned comparisons showed that up to the third session, the three conditions significantly differed before they started to merge. Although the difference between the Cover and No Cover conditions tended to be smaller than in Experiment 1, in Session 1, which was uncontaminated by further extinction, there was again a clearly significant difference (*p* = 0.008). Moreover, the Cover condition was also significantly different from the Acquisition Control condition in Session 1 (*p* = 0.012). Thus, although the cover again protected from complete extinction in the Test phase, it did not fully abolish extinction relative to the Acquisition Control condition. This middle position makes sense since the cover removed access to information about the status of the outcome event. Because either the presence or the absence of sucrose was possible states of the outcome when the receptacle was covered, the intermediate response levels observed in the Cover group seem rational.

## Experiment 3

The previous experiments have shown that the presence of a cover blocking informational access to the receptacle during the Extinction phase led to diminished extinction during the Test phase. On a cognitive account, this can be interpreted as evidence for rats’ sensitivity to the difference between explicitly absent outcomes versus lack of evidence about the status of the outcomes. Alternatively, a context change theory might interpret the findings as evidence for a renewal effect. According to this theory, the switch from a metallic shield with an opening during magazine training and acquisition to a metallic shield without an opening might constitute a salient context change that could explain why expectancies of sucrose were again high in the Test phase in the Cover condition.

The goal of Experiment 3 was to provide a stronger test of these two competing theories. In Experiment 3, in all conditions, no metallic shields were presented during magazine training and the Acquisition phase. Thus, in both experimental conditions, Cover and No Cover, we introduced an intact metallic plate in the Extinction phase, which, on a renewal account, should in both cases qualify as context change. The crucial manipulation involved the location of the cover during the Extinction phase. Whereas in the Cover condition, the metallic plate was, as in previous experiments, placed over the drinking receptacle, thus obstructing access to information about presence or absence of sucrose, in the No Cover condition, we placed the intact metallic plate slightly left of the receptacle where it did not obstruct access. Thus, in both conditions, the same metallic plate was introduced in an equally visible position, which should therefore lead to a renewal effect relative to an appropriate control condition. By contrast, on our cognitive account, rats should differentiate between the condition in which relevant information is inaccessible (Cover) and the condition in which information about the absence of sucrose is accessible (No Cover). If this, and not context change drives the predicted effect, we would expect that the No Cover condition would show similarly low expectancies in the Test phase as a regular extinction condition without any metallic plates. Such a condition served as our Control condition.

### Subjects and procedure

Twenty-four experimentally naïve female Long-Evans rats served as subjects, which were randomly assigned to three groups (Cover, No Cover, and Control). The equipment and procedure were the same as in Experiment 1 except where noted below. As in the previous experiments, all rats received 60 min of magazine training with identical ITIs. In contrast to the previous experiments, no metallic plates were present. Phase 1 Acquisition training was as in Experiment 2 except that, due to inexplicably low performance by Session 6 in this experiment, we extended learning to 10 days to establish the learning preconditions for the following phases. Unlike in the previous studies, no metallic plates were present throughout this phase. As in the previous experiments, learning and extinction were measured using discrimination indices (i.e., difference scores), which compared nose pokes. Phase 2, Extinction, progressed for three days like in Experiment 1. In the Cover condition, a metallic plate prevented access to the drinking receptacle, as in the previous experiments. By contrast, in the new No Cover condition, the cover was intact but moved slightly to the left of the receptacle, rendering the drinking receptacle completely unobstructed. In the Control condition, rats received conventional extinction trials, in which no plates were present in the chambers. On each day, all subjects received 12 extinction trials with empty dippers in each 30-min session (ITIs as in previous studies). Nose pokes served as indicators of expectancies in the two applicable conditions (No Cover and Control). During the Test phase, no metal plates were present in the chambers. Otherwise, testing was similar to the testing procedure used in the previous experiments (i.e., three trials per 30 min). We recorded all nose pokes during a 30-s pre-CS interval and during the presentation of each CS. No sucrose was delivered during these sessions. As in the previous studies, we tested rats for six days.

### Results and discussion

**Fig. 4:**
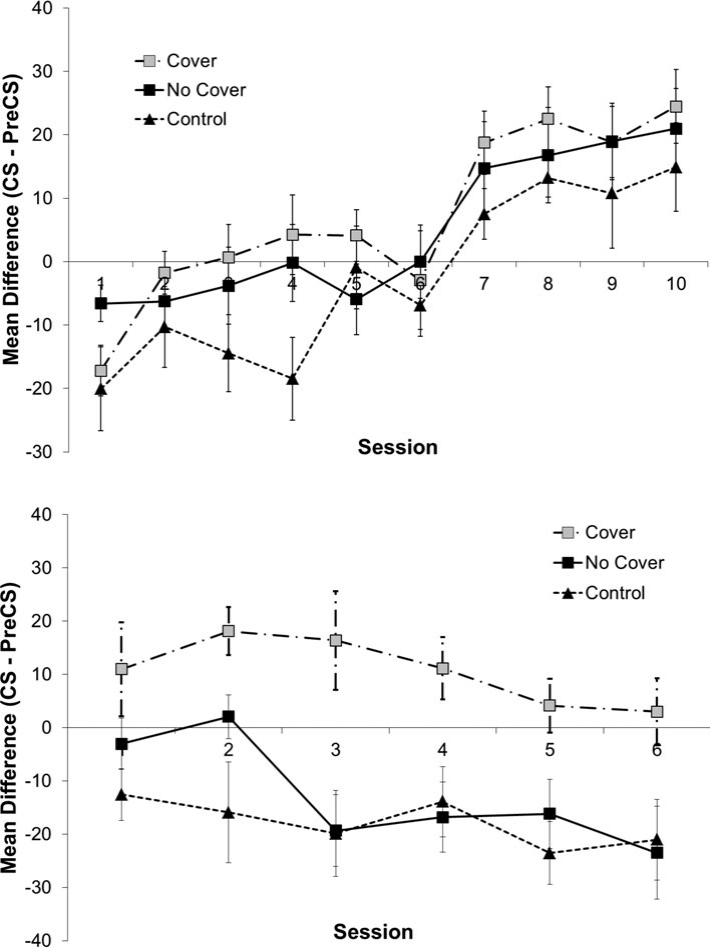
(*Top panel*) Mean difference (CS—pre-CS) scores (discrimination index) as a function of Phase 1 Acquisition session for the Cover, No Cover, and Control conditions in Experiment 3. *Error bars* represent standard errors. (*Bottom panel*) Mean difference (CS—pre-CS) scores (discrimination index) as a function of Phase 3 Test session for the Cover, No Cover, and Control conditions in Experiment 3. *Error bars* represent standard errors

One subject from the Control condition failed to learn the contingency and was therefore dropped from the analyses (i.e., *n* = 7 in Control). In each of the two other conditions, 8 subjects participated successfully. Figure 4 (top panel) shows the results from the Acquisition phase, as measured by discrimination indices (difference score). The analysis revealed a main effect of Session, *F*(5.15,102.98) = 15.53, *p* < 0.001, MSE = 394.6, which indicates that all remaining subjects had successfully acquired knowledge about the contingency between light and sucrose after the 10 sessions. There was no significant main effect of condition, *F*(2, 20) = 2.53, *p* = 0.104, MSE = 733.8, nor a significant interaction (*F* < 1).

The most interesting analyses concern the Test phase. Again we analyzed discrimination indices (difference score). Not surprisingly, the analysis revealed a main effect of Test Session, *F*(5, 100) = 3.10, *p* = 0.012, MSE = 252.965, signifying additional extinction during the test trials. Most importantly, the analysis revealed a main effect of condition, *F*(1, 20) = 12.130, *p* < 0.001, MSE = 878.874. There was no significant interaction, *F* < 1. Since there was no significant interaction, planned comparisons were conducted comparing each group against every other group collapsed across all sessions. A significant difference was found between the Cover condition and both the No Cover and the Control conditions (*p* = 0.001 and *p* < 0.001, respectively). No significant difference was found between the No Cover and Control conditions (*p* = 0.434), however.

In sum, the results show that the presence of a cover again partially preserved the learned contingency from extinction in the test trials, whereas extinction was equally strong regardless of whether a cover was newly introduced next to the receptacle (No Cover), or no cover was presented throughout the experiment (Control). The lack of a difference between the No Cover and Control conditions indicates that the added presence of a metallic plate in the chambers did not constitute a relevant context change that would lead to renewal. Only if the cover had a specific position, making it uncertain for the learner whether the outcome was absent or present (behind the cover) was behavior observed that indicated that the rats continued to expect the outcome behind the occluder. In the next section, we will discuss the relevance of this finding for the two competing theories.

## General discussion

Coordinating contingency information with representations of the underlying causal texture of the world is an important competency of intelligent organisms. Our sensory input does not always faithfully reflect causal relations and is therefore ambiguous. Our goal in the present experiments was to study whether rats display in their behavior sensitivity to this ambiguity.

We used a conventional Pavlovian extinction paradigm to confront subjects with changes in experienced contingencies. Whereas in the Acquisition phases, rats experienced a perfect contingency between light signals and sucrose, in the Extinction phases, the light signals were paired with the absence of perceivable sucrose. The crucial manipulation involved the presentation of the absent outcome. Whereas in the Cover conditions, access to the drinking receptacle was covered by an intact metal plate, in different variants of the No Cover conditions, the drinking receptacle was accessible. The results in the Test phase showed that this manipulation influenced expectancies (i.e., nose pokes) in the Test phases. In the Cover conditions, the measured expectancies of sucrose were clearly at a higher level in the Test phase than in the No Cover conditions.

We have discussed two theories competing to explain our findings. On our cognitive account, the observed difference is consistent with the interpretation that the rats were able to understand that the cover blocked informational access to the outcome. Thus, they might have inferred that sucrose might still be contingent on light signals, although it was presently not observable. In contrast, in the No Cover conditions, the experienced absence of sucrose was plausibly interpreted as signaling a change in the underlying contingency.

An alternative associationist account, renewal theory, attributes differences in the nose pokes in the Test phase to the presence or absence of context changes (see Bouton 2004; Delamater 2004; Pearce and Bouton 2001). This theory does not require sophisticated cognitive distinctions between objects and available information, but rather assumes that rats simply register visible changes in the stimuli being part of the learning context. On this account, in Experiments 1 and 2, in the Cover conditions, the metal plate looked different during Phase 2 Extinction than during the other phases, which might have led to a renewal effect consistent with the observed behavior. By contrast, the metal plate remained unmodified across all three phases in the No Cover conditions. Hence, no renewal was observed, according to this theory.

To provide stronger evidence for our cognitive account, we designed Experiment 3, in which there was a similar context change in both the Cover and the No Cover conditions. In both conditions, an intact metallic plate was introduced in the chambers during the Extinction phase, whereas in the previous magazine training, and the Acquisition and Test phases, no metallic plate was present. The crucial difference between the conditions was the placement of the cover: Whereas in the Cover condition, the metallic plate again blocked access to the drinking receptacle, in the No Cover condition, it was placed slightly to the left of the receptacle. Thus, a renewal effect should be observed in both experimental conditions. However, as in the other experiments, we found a significant difference between the Cover and No Cover conditions in the Test phase.

It is not possible to completely disprove the renewal account because it always can be adapted post hoc to the data. The key question is whether such an effect can be *predicted* with the theoretical resources of an associationist theory that denies rats representations about hidden states. It is unclear how such a prediction could be derived, unless the theorist smuggles in her own cognitive intuitions about the task.

### Further evidence for cognitive representations

The reported studies are part of an ongoing series, in which we investigate the cognitive competencies of rats (Blaisdell et al. 2006, 2009; Fast and Blaisdell 2011; Leising et al. 2008). The present results reveal competencies that go beyond the representational power of most associative learning theories. The experiments show that rats do not only code presence or absence of learning events, but appreciate that absence of an experience may not necessarily be correlated with the absence of the underlying event. Most modern learning theories acknowledge that rats (in contrast to non-mammalian vertebrates) generate expectancies in both classical and instrumental conditioning, which may be either confirmed or frustrated (Colwill and Rescorla 1986; Papini 2006). Thus, it does not seem far-fetched to attribute cognitions to rats that go beyond simple registrations of context features. It is by now a very well established fact that rats form and test expectancies during observational and instrumental learning. Our research goes beyond these findings, however. Confirmation or frustration is only possible on the basis of learning input. This learning input is ambiguous since it may signal presence, absence, or alternatively lack of evidence. Our experiments show that rats seem to appreciate this difference, which allows them to flexibly coordinate sensory input with the representations of the underlying events.

One interesting feature of research on animal cognition is the historical connections between types of theories and species. Whereas rats have typically been studied in the context of associative learning theories that are historically skeptical when it comes to cognitive interpretations (see extinction theories), researchers have been more open to these kinds of theories with other species (e.g., primates and dolphins). The ability to find hidden objects may be such a pervasive function for successful adaptations that its cognitive precursors may have been part of the competencies of various species from early on. Thus, our finding that rats are capable of interpreting the significance of their learning input may be less surprising than initially thought. Instead, it may be a natural byproduct of the adaptive goal to obtain stable representations of the causal texture of the world.
